# Biosynthesis of Metal and Metal Oxide Nanoparticles Using Microbial Cultures: Mechanisms, Antimicrobial Activity and Applications to Cultural Heritage

**DOI:** 10.3390/microorganisms11020378

**Published:** 2023-02-02

**Authors:** António Carrapiço, Maria Rosário Martins, Ana Teresa Caldeira, José Mirão, Luís Dias

**Affiliations:** 1 HERCULES Laboratory, Cultural Heritage, Studies and Safeguard, University of Évora, 7000-809 Évora, Portugal; 2 Institute for Research and Advanced Training (IIFA), University of Évora, 7000-809 Évora, Portugal; 3 Department of Medicinal Sciences and Health, School of Health and Human Development, University of Évora, 7000-671 Évora, Portugal; 4 Department of Chemistry and Biochemistry, School of Sciences and Technology, University of Évora, 7000-671 Évora, Portugal; 5 Department of Geosciences, School of Sciences and Technology, University of Évora, 7000-671 Évora, Portugal

**Keywords:** green synthesis, NPs, metallic nanoparticles, metal-based nanoparticles, properties, nanotechnology, preservation

## Abstract

Nanoparticles (1 to 100 nm) have unique physical and chemical properties, which makes them suitable for application in a vast range of scientific and technological fields. In particular, metal nanoparticle (MNPs) research has been showing promising antimicrobial activities, paving the way for new applications. However, despite some research into their antimicrobial potential, the antimicrobial mechanisms are still not well determined. Nanoparticles’ biosynthesis, using plant extracts or microorganisms, has shown promising results as green alternatives to chemical synthesis; however, the knowledge regarding the mechanisms behind it is neither abundant nor consensual. In this review, findings from studies on the antimicrobial and biosynthesis mechanisms of MNPs were compiled and evidence-based mechanisms proposed. The first revealed the importance of enzymatic disturbance by internalized metal ions, while the second illustrated the role of reducing and negatively charged molecules. Additionally, the main results from recent studies (2018–2022) on the biosynthesis of MNPs using microorganisms were summarized and analyzed, evidencing a prevalence of research on silver nanoparticles synthesized using bacteria aiming toward testing their antimicrobial potential. Finally, a synopsis of studies on MNPs applied to cultural heritage materials showed potential for their future use in preservation.

## 1. Introduction

Since ancient times, humans have unknowingly used and manipulated nanoparticles (NPs) for several applications. One of the oldest known applications of NPs goes back to the 4th century with the manufacturing of the Lycurgus Cup [1] whose visual properties (green upon light reflection, red upon light transmittance) are derived from the presence of silver and gold nanoparticles (AgNPs and AuNPs). However, despite being manufactured in ancient times, it was not until 1990 that this explanation was ascertained using transmission electron microscopy (TEM) [2]. The idealization of knowingly manipulating NPs using technology is attributed to Richard Feynman based on a lecture titled “There’s Plenty of Room at the Bottom” in 1965. However, the first actual use of nanotechnology was only achieved in 1980 by Norio Taniguchi [3]. Today, nanotechnology is described as a science that studies and manipulates particles in the order of 10^−9^ m, with sizes ranging from 1 to 100 nm [4]. Being relatively new, this field of science has been growing since its birth, with applications in a vast number of scientific areas. In particular, the application of nanotechnology for the study of metal nanoparticles (MNPs) has experienced significant growth in the last two decades. Nanoparticles are usually divided into three categories: organic nanoparticles (e.g., liposomes), carbon-based nanoparticles (e.g., fullerenes) and metal-based nanoparticles (MNPs) (e.g., metal oxide nanoparticles) [5]. The latter are composed of a metal core usually covered with a shell, which may be of inorganic or organic origin [6]. Based on their elemental composition, metal-based nanoparticles can be subdivided into metal oxides, metal sulfides, metal organic frameworks, doped metal/metal oxides and metal nanoparticles [7]. MNPs are especially interesting due to properties such as optical polarizability, electrical conductivity, photocatalysis and antimicrobial activity [8], which makes them useful for many applications in areas from electronics to pharmaceutics [8].

One of the most studied properties of MNPs is their antimicrobial activity [9], especially due to their potential use against multi-drug-resistant microorganisms (MDRM) [10]. In addition to this, their relatively easy functionalization, which further facilitates their applications and manipulation (e.g., through immobilization), also contributes to the interest in studying and trying to develop functionalized MNPs with enhanced properties. There are already several commercially available products containing MNPs [11]. However, most MNPs are synthesized using chemical methods that produce toxic by-products [12]; hence, their production is an environmental concern. Fortunately, in the last decade, the number of studies trying to develop environmentally safer synthesis methods has been growing. In particular, it has been proven that both microorganisms (i.e., intracellular synthesis) and molecules secreted by them (i.e., extracellular synthesis) can be used to safely synthesize MNPs [13]. Both intracellular and extracellular synthesis are generically called biosynthesis. Even so, since intracellular synthesis requires laborious isolation methods, which needlessly consume both time and resources, extracellular biosynthesis is more promising. For this reason, only extracellular biosynthesis will be discussed here.

Biosynthesis of MNPs is a promising alternative to chemical synthesis because of its apparently lower environmental impact [14]. Additionally, MNPs (extracellular) biosynthesis can usually be achieved in “one pot” reactions at room temperature. On the other hand, their functionalization occurs simultaneously with their synthesis through their capping with organic molecules from the reaction media, which aids their antimicrobial activity and increases their stability [9]. However, the mechanisms of both biosynthesis and antimicrobial activity of these MNPs are not fully ascertained.

As the UNESCO reminds us, “(…) Our cultural and natural heritage are both irreplaceable sources of life and inspiration” [15]. Therefore, it is our responsibility to preserve it for future generations to study, learn from and enjoy. One of the problems that lead to cultural heritage loss and degradation is microbial contamination, which is commonly promoted by physical damages associated with exposure to environmental conditions (e.g., humidity, temperature, light exposure, wind exposure) [16,17,18]. However, due to their usually sensitive nature, the use of physical methods and the application of chemicals on these materials may lead to undesired effects (e.g., abrasion and chemical leaching), which may themselves contribute to their deterioration [16]. Because of their apparently lower environmental toxicity, higher stability and relatively easy immobilization and synthesis, in the last decade, the application of MNPs to cultural heritage material has been tested, and some information has been acquired and published. Despite some reviews having been written recently [19,20,21], the ever-growing nature of this field requires the new scattered information to be compiled to facilitate its consultation by researchers, consequently increasing knowledge dissemination and improving research productivity.

In this review, studies on the mechanisms of MNPs extracellular biosynthesis—using mostly microorganisms’ growth media supernatants—were compiled. Information regarding the techniques used in these studies as well as their individual findings are presented. Based on the analysis of observations from all these studies, a possible mechanism was proposed. Then, a compilation of studies from the last five years (2018–2022) was produced. Information regarding the microorganisms used, the MNPs obtained, their size and their studied properties were summarized. Afterward, published research on the study of the antimicrobial mechanism of MNPs was also collected and an integrated mechanism hypothesized. Additionally, studies regarding the use of MNPs for antimicrobial applications on materials used in cultural heritage objects and buildings were described for each available material (stone, paper, textiles and wood). Studies where MNPs antimicrobial activity was determined against microorganisms isolated from cultural heritage materials were also highlighted. Moreover, information on the material, method and ascertained properties of MNPs was also summarized. Finally, some guidelines for future research were proposed.

## 2. Metal Nanoparticles Synthesis Using Biological Extracts

It is known that it is possible to synthesize MNPs by adding metal salts to both plant extracts and cell-free supernatants of liquid microbial cultures. However, despite being similar, there are little variations in the biosynthesis of different MNPs. For instance, different precursors lead to MNPs with distinct characteristics [22], while variations in the concentration of elements, such as molecular oxygen (O_2_) or chloride (Cl^−^), may result in the formation of metal oxide nanoparticles (e.g., Ag_2_ONPs) or metal chloride nanoparticles (e.g., AgClNPs) instead of metal nanoparticles (e.g., AgNPs) [23]. Moreover, it has also been shown that several reaction conditions, such as temperature, oxygenation, pH, precursor (metal salt) concentration, microbial growth phase (upon supernatant collection), incubation time and irradiation, highly influence both the yield of the reaction and the properties of the MNPs [24,25,26]. However, the mechanism behind these phenomena is not yet fully understood. Notwithstanding, several studies using plant extracts and microorganisms’ cell-free supernatants have been performed in an attempt to identify the molecules responsible for the reduction and stabilization (capping agents) of these NPs [27,28,29,30,31,32,33,34,35,36,37,38,39,40,41,42,43,44,45,46]. In these studies, several techniques have been applied. 

### 2.1. Techniques Employed in the Study of Metal Nanoparticles’ Biosynthesis Mechanisms

Aiming toward the identification of the functional groups that may be involved in the reactions, Fourier transform infrared spectroscopy (FTIR) analysis of the reaction media before and after synthesis has been the most used technique [27,28,29,30,31,32,33,35,41]. X-ray photoelectron spectroscopy (XPS) was also used for the same purpose [29]. One thermogravimetric study was also conducted [27]. Chromatographic techniques, such as gas chromatography (GC) [29], high-performance liquid chromatography (HPLC) [32], ultra-high-performance liquid chromatography (UPLC) [34], liquid chromatography (LC) [40] and gel permeation chromatography (GPC) [41], have been employed to separate and identify the several compounds present in the reaction solutions before and after synthesis. The latter are usually followed by identification techniques, such as mass spectrometry (MS) [29,40] and high-resolution mass spectrometry (HRMS) [34]. One study also used matrix-assisted laser desorption/ionization time-of-flight mass spectrometry (MALDI-TOF-MS), aiming to identify the capping agents of MNPs [39]. Separation of proteins present in the media using sodium dodecyl sulfate–polyacrylamide gel electrophoresis (SDS-PAGE) has also been performed [40] to try to prove their role in the reaction, as well as to determine their molecular weight. Quantifications of several compounds in both the media and nanoparticle suspensions were performed to understand their role in the reactions, as well as their relative contribution to the synthesis—e.g., DTNB, DNSA, DTNP and Folin-phenol assays [40,41]. Spectrophotometric analysis of the NPs plasmon peak associated with changes in the reaction media was generally performed [27,36,37,38,40,41,42] with the assumption that higher absorbance values are related to increases in concentration, and the shifts in the peak toward higher wavelengths (toward red) are due to the increase in the diameter of the nanoparticle [47]. Finally, cyclic voltammetry was used to try to prove the effective role of specific compounds (i.e., caffeine) in the reduction in metal ions [32]. The analysis of the data acquired from these techniques enabled researchers to propose mechanisms for nanoparticle synthesis using biological extracts (i.e., plant extracts and cell-free supernatant of microorganism cultures).

### 2.2. Biosynthesis Mechanisms—State of the Art

It has initially been hypothesized that MNPs synthesis using cell-free supernatants of microorganisms was achievable due to the secretion of enzymes responsible for the reduction in compounds bound to the metal—e.g., reduction in nitrate from silver nitrate using nitrate reductase [36,37,39]. However, this hypothesis has been rebutted by other studies that show the role of other molecules (e.g., reducing sugars [40], nitrogenous biomolecules (e.g., proteins) [35,38,40,41,42], GSH [40,42], NADH and NADPH [40,42], polysaccharides [41], glycoproteins [41] and proteoglycans [41]). One study, aiming to ascertain the role and relative contribution of enzymes to the synthesis of NPs, used the cell-free denatured protein fraction of microorganism culture supernatant to prove that, despite contributing to the synthesis of NPs, enzyme catalysis is not mainly responsible or even required [38]. Moreover, studies on plant extracts that also led to the synthesis of MNPs evidenced the role of several molecules in the reduction (reducing sugars [27], flavonoids [27,28,34], proteins [27], polysaccharides [27], aldehydes [28], phenolic compounds [29,34] and alkaloids [32]) and stabilization (capping) (reducing sugars [27], flavonoids [27,28,29], phenolic compounds [28,34], alcohols [29], amines [29], alkanes [29] and alkaloids [32]) of MNPs, some of which are also present in cell-free supernatants of microorganism culture media [48,49,50]. The synthesis of MNPs using isolated compounds, such as cysteine [43], flavonols (DMY) [44], caffeic acid [45] and alginate [46], also proved that enzymatic catalysis is not mandatory for metal nanoparticle synthesis. 

### 2.3. Evidence-Based Proposed Biosynthesis Mechanisms

Several studies referenced above used plant extracts and cell-free supernatants of microorganism cultures to synthesize MNPs (plant extracts—Au, Ag, Cu, Fe and Zn; microorganisms—Ag and Au) aiming toward the study of the molecules involved and proposal of synthesis mechanisms. 

**Figure 1:**
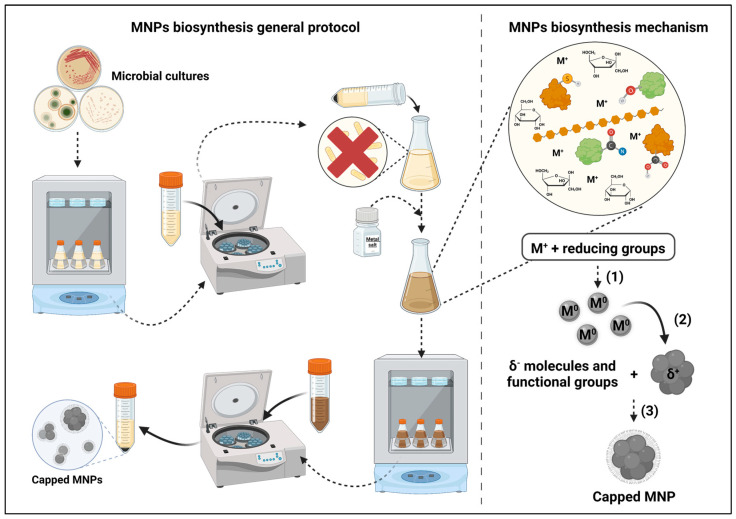
Metal nanoparticles biosynthesis’ mechanisms and general protocol. (1) Metal reduction by enzymes and other molecules from the supernatant; (2) reduced metal particle agglomeration; (3) MNPs capping by negatively charged molecules from the supernatant. Created with BioRender.com.

From these studies, some general principles were evidenced regarding the possible mechanisms that lead to metal nanoparticle synthesis using the cell-free supernatant of microorganism cultures (Figure 1). Firstly, the molecules detected in the reaction media, as well as their relative abundances, seem to be highly influenced by both the microorganisms used and the composition of the growth media. Secondly, the role of secreted enzymes, despite being evidenced by several studies [36,37,39,42], is not overwhelming when compared with other molecules or mandatory for synthesis to occur. Notwithstanding, enzymatic catalysis seems to contribute to the increase in the reaction speed.

There seems to be strong evidence that the sole requirement for metal nanoparticle synthesis to occur is the presence of molecules with reducing groups, such as carboxyl, amide, thiol and hydroxyl. The latter seems to be of great importance given its presence in most molecules, which have been determined to play a role in metal reduction, and based on the evidence of the reduction in its signal after synthesis in FTIR analysis of the reaction media. Nevertheless, despite further research being required, the effect of the presence of reducing enzymes on the speed of reaction seems to be relevant. However, given that most of the studies do not report the yield of the reaction, it is impossible to accurately compare syntheses with different reaction conditions. Reporting that the synthesis occurred based solely on spectrophotometric analysis and microscopy (e.g., scanning electron microscopy (SEM) and transmission electron microscopy (TEM)) does not give enough information to hypothesize about the real importance of the different compounds present in the reaction media. On the other hand, the presence of negatively charged groups (e.g., carbonyl, amine) or atoms (e.g., nitrogen) seems to be indispensable for the adsorption of capping molecules to the MNPs, which are responsible for their stabilization and influence their antimicrobial activity [9]. However, analogously to the yield, not all studies determine the antimicrobial properties of the NPs, which does not enable the determination of the impact of the different capping molecules on these properties.

## 3. Recent Studies on the Biosynthesis of Metal Nanoparticles Using Microorganisms

An analysis of 149 different research articles from the last 5 years (2018 to 2022) where MNPs (metal, metal oxide, metal chloride and metal sulfide) were obtained using 72 different species of 5 microorganism classes (44 bacteria, 1 archaeon, 20 fungi (14 molds and 6 yeasts) and 7 microalgae) was conducted (Table 1, Table 2 and Table 3).

Despite fungi cell-free supernatants seemingly resulting in NPs with smaller diameters (Table 2), syntheses using bacteria cell-free supernatants were prevalent among the studies compiled in this work (bacteria 55%, fungi 39% (molds 34%, yeasts 5%), microalgae 5%, archaea 1%). Given that smaller NPs seem to be associated with better antimicrobial properties [135], this observation might be linked to the methodologies that are usually employed in each of these microorganism classes. Usually, bacteria cell-free supernatants are obtained from centrifugation of the culture media and used directly in the synthesis of NPs, while most cell-free supernatants from molds are obtained following a period of incubation—after growth in liquid culture media—in distilled water or buffer. Moreover, the incubation time needed for the growth of fungi (72 h to 96 h) is usually longer than for bacteria (24 h to 48 h). These circumstances might help explain the preference for studies using bacteria rather than fungi. 

Increased complexity in culture growth and nanoparticle synthesis might also explain the lower number of syntheses using cell-free supernatant of microalgae (5%) (Table 3), which, similarly to fungi, also seem to result in smaller NPs.

**Table 2:** Biosynthesis of metal nanoparticles using fungi. Several examples of literature published in the last five years (2018–2022). The microbial genera, the metal and its precursor and the size of the metal nanoparticles obtained are presented. It is also stated whether the antimicrobial activity and toxicity of the nanoparticles were tested and which properties were found.

Metal *	Microbial Genera	NPs Size (nm)	Precursor	Antimicrobial Activity Studies	Toxicity Studies	Main Properties	Ref.
Molds							
Ag	*Anamorphous*	10 to 70	AgNO_3_	Yes	Yes	Antimicrobial	[136]
Ag	*Aspergillus*	1 to 50	AgNO_3_	Yes	No	Antimicrobial	[137]
Ag	*Aspergillus*	3 to 28	AgNO_3_	Yes	Yes	Antimicrobial Photocatalytic Acaricidal	[138]
Ag	*Aspergillus*	5 to 37	AgNO_3_	Yes	No	Antimicrobial	[139]
Ag	*Aspergillus*	15 to 35	AgNO_3_	No	No	NS	[140]
Ag	*Aspergillus*	7 to 23	AgNO_3_	Yes	Yes	Antimicrobial Cytotoxicity	[141]
Ag	*Aspergillus*	13 to 49	AgNO_3_	Yes	Yes	Antimicrobial Cytotoxicity	[142]
Ag	*Aspergillus*	1 to 21	AgNO_3_	Yes	Yes	Antimicrobial Cytotoxicity	[143]
Ag	*Aspergillus*	3.5 to 28.2	AgNO_3_	Yes	No	Antiamoebic	[144]
Ag	*Aspergillus*	~100 ^a,b^	AgNO_3_	Yes	Yes	Antimicrobial Cytotoxicity	[145]
Ag	*Aspergillus*	1 to 10.5	AgNO_3_	Yes	No	Antimicrobial	[66]
Ag	*Aspergillus*	2 to 13		No	Yes	Mosquitocidal	[146]
Ag	*Aspergillus*	56 ^a,b^		Yes	No	Antimicrobial	[147]
Ag	*Aspergillus*	20 to 60	AgNO_3_	Yes	No	Antimicrobial Antioxidant Photocatalytic	[60]
Ag	*Aspergillus*	10 to 100	AgNO_3_	Yes	Yes	Antimicrobial Cytotoxicity	[148]
Ag	*Aspergillus*	1 to 15	AgNO_3_	Yes	No	Antimicrobial Antioxidant	[149]
Ag	*Botryodiplodia*	66.8 to 111.2	AgNO_3_	No	Yes	Cytotoxicity	[150]
Ag	*Eurotium*	15 to 20	AgNO_3_	Yes	No	Antimicrobial	[151]
Ag	*Fusarium*	~40 ^c^	AgNO_3_	Yes	No	Antimicrobial Photocatalytic	[152]
Ag	*Fusarium*	2 to 20	AgNO_3_	Yes	No	Antimicrobial	[153]
Ag	*Humicola*	15 to 40	AgNO_3_ Na_2_SO_3_	No	Yes	Antiparasitic Cytotoxicity	[154]
Ag	*Letendraea*	33.8 ^a^	AgNO_3_	No	Yes	Photocatalytic Antialgal	[155]
Ag	*Letendraea*	8 to 56	AgNO_3_	Yes	No	Antimicrobial Antioxidant Photocatalytic	[156]
Ag	*Neopestalotiopsis*	4.8 to 20.7	AgNO_3_	Yes	Yes	Antimicrobial Antibiofilm	[157]
Ag	*Penicillium*	2 to 20	AgNO_3_	Yes	No	Antimicrobial	[158]
Ag	*Penicillium*	18 to 60	AgNO_3_	Yes	No	Antimicrobial	[159]
Ag	*Penicillium*	48.2 ^a,b^	AgNO_3_	Yes	No	Antimicrobial Antibiofilm	[160]
Ag	*Penicillium*	60 to 80	AgNO_3_	Yes	No	Antimicrobial	[161]
Ag	*Phomopsis*	5 to 60	AgNO_3_	Yes	No	Antimicrobial	[162]
Ag	*Talaromyces*	5 to 30	AgNO_3_	Yes	Yes	Antimicrobial Cytotoxicity Larvicidal	[163]
Ag	*Trichoderma*	10 to 70	AgNO_3_	Yes	Yes	Antimicrobial Antibiofilm Antioxidant Cytotoxicity	[164]
Ag	*Trichoderma*	5 to 35	AgNO_3_	Yes	No	Antimicrobial	[165]
Ag	*Trichoderma*	5 to 50	AgNO_3_	Yes	No	Antimicrobial	[166]
Ag	*Trichoderma*	15 to 25	AgNO_3_	No	No	NS	[167]
Au	*Aspergillus*	37 to 62	HAuCl_4_	Yes	No	Antimicrobial	[139]
Au	*Aspergillus*	20 to 50	AuCl_3_	No	No	NS	[140]
Au	*Aspergillus*	30 to 40	AuCl_3_	Yes	No	Antimicrobial Antibiofilm	[168]
Au	*Aspergillus*	7 to 15	HAuCl_4_	No	Yes	Photocatalytic Cytotoxicity	[169]
Au	*Fusarium*	22 to 30	HAuCl_4_	Yes	No	Antimicrobial	[170]
Au	*Trichoderma*	8 to 30	HAuCl_4_	Yes	Yes	Antimicrobial Antibiofilm Antioxidant Cytotoxicity	[164]
Au	*Trichoderma*	1 to 24	HAuCl_4_	No	No	Photocatalytic	[171]
Cu	*Aspergillus*	9 to 25	CuSO_4_	No	No	NS	[172]
Cu	*Penicillium*	10.5 to 59.7	Cu(CH_3_COO)_2_	Yes	No	Antimicrobial Antibiofilm	[173]
Cu	*Trichoderma*	1.3 to 30	CuSO_4_	Yes	Yes	Antimicrobial Cytotoxicity	[174]
Fe	*Aspergillus*	6.0 to 36.0	FeCl_3_	No	No	Photocatalytic Detoxification	[175]
Fe	*Aspergillus*	32.7 to 47.6	FeSO_4_	Yes	No	Antimicrobial Photocatalytic	[176]
Fe	*Aspergillus*	73.1 ^a^	Fe(NO_3_)_3_	No	No	Detoxification	[177]
Fe	*Penicillium*	15 to 66	FeCl_3_	No	No	Photocatalytic	[178]
Mg	*Aspergillus*	20.0 to 86.0	Mg(NO_3_)_2_	No	No	Photocatalytic Detoxification	[175]
Mg	*Aspergillus*	30 to 85	Mg(NO_3_)_2_	Yes	Yes	Photocatalytic Detoxification	[179]
Mg	*Aspergillus*	8 to 38	Mg(NO_3_)_2_	Yes	No	Antimicrobial Photocatalytic Detoxification	[180]
Mg	*Penicillium*	7 to 40	Mg(NO_3_)_2_	Yes	No	Antimicrobial Mosquitocidal	[181]
Mg	*Rhizopus*	8.0 to 47.5	Mg(NO_3_)_2_	Yes	No	Antimicrobial Mosquitocidal Photocatalytic Detoxification	[182]
Pt	*Penicillium*	2 to 25	H_2_PtCl_6_	Yes	Yes	Antimicrobial Cytotoxicity	[183]
V	*Fusarium*	10 to 20	NH_4_VO_3_	Yes	Yes	Antimicrobial Cytotoxicity	[184]
Zn	*Aspergillus*	10 to 45	Zn(CH_3_CO_2_)_2_	Yes	Yes	Antimicrobial UV protection	[185]
Zn	*Aspergillus*	80 to 100 ^a^	Zn(CH_3_CO_2_)_2_	Yes	No	Antimicrobial Photocatalytic Antibiofilm	[186]
Zn	*Cochliobolus*	2 to 9	Zn(CH_3_CO_2_)_2_	No	No	Photocatalytic	[187]
Zn	*Cochliobolus*	2 to 6	Zn(CH_3_CO_2_)_2_	No	No	NS	[188]
Zn	*Penicillium*	9 to 35	Zn(CH_3_CO_2_)_2_	Yes	No	Antimicrobial Antibiofilm	[173]
Yeasts							
Ag	*Candida*	2.7 ^d^	AgNO_3_	Yes	No	Antimicrobial	[189]
Ag	*Pichia*	4 to 12	AgNO_3_	Yes	Yes	Antimicrobial Antioxidant Cytotoxicity Photocatalytic	[190]
Ag	*Pichia*	20 to 30	AgNO_3_	Yes	Yes	Antimicrobial Anti-inflammatory Cytotoxicity	[191]
Ag	*Saccharomyces*	11 to 25	AgNO_3_	Yes	No	Antimicrobial	[192]
Ag	*Saccharomyces*	7.3 ^d^	AgNO_3_	Yes	No	Antimicrobial	[189]
Ag	*Saccharomyces*	12 to 21	AgNO_3_	Yes	Yes	Antimicrobial Anti-inflammatory Cytotoxicity	[191]
Ag	*Yarrowia*	50 ^a^	AgNO_3_	No	No	Antimicrobial	[193]
Au	*Magnusiomyces*	20 to 30	HauCl_4_	No	No	Photocatalytic	[194]
Pt	*Rhodotorula*	2.83 ^a^	H_2_PtCl_6_	Yes	No	Antimicrobial Antioxidant	[195]

* Metal element from the obtained nanoparticles: in metal oxide nanoparticles and other nanoparticles (e.g., chloride or sulfide), the non-metallic elements are omitted (e.g., O, Cl, S). NS: not studied; ^a^ Mean value; ^b^ Measured with dynamic light scattering (DLS); ^c^ Most nanoparticles; ^d^ Measured with atomic force microscopy (AFM).

Regarding the metals used to synthesize NPs, there are also clear preferences. Silver is by far the most studied and reported metal (63%), followed by copper (9%), zinc (9%), gold (8%) and iron (6%). Magnesium (3%), platinum (1%), titanium (1%) and vanadium (1%) were also studied. Nano-silver multifunctional properties [196] and their applications in different fields associated with the fact that most commercially available products are chemically synthesized [11], which results in highly toxic and pollutant waste products [197], might be associated with these numbers. Given that the synthesis of MNPs using cell-free supernatant of microorganisms is considered “green” because it does not require highly toxic or pollutant chemicals [197], there might be an incentive to preferably study this metal to find environmentally friendly synthesis alternatives to be applied in the already established industry and market. Moreover, despite AuNPs presenting similar properties [198], the economic cost associated with their synthesis can deter researchers from studying it. The same could be said for platinum. Other MNPs, such as copper, iron and zinc, do not present the same properties as silver, gold or platinum, being less stable and having less effective antimicrobial and photocatalytic properties [198].

**Table 3:** Biosynthesis of metal nanoparticles using microalgae. Several examples of literature published in the last five years (2018–2022). The microbial genera, the metal and its precursor and the size of the metal nanoparticles obtained are presented. It is also stated whether the antimicrobial activity and toxicity of the nanoparticles were tested and which properties were found.

Metal *	Microbial Genera	NPs Size (nm)	Precursor	Antimicrobial Activity Studies	Toxicity Studies	Main Properties	Ref.
Microalgae							
Ag	*Chlorella*	5.3 ^a,c^	AgNO_3_	Yes	No	Antimicrobial	[189]
Ag	*Chlorella*	10 to 20 ^b^	AgNO_3_	Yes	No	Antimicrobial	[199]
Ag	*Lyngbya*	10 to 20 ^b^	AgNO_3_	Yes	No	Antimicrobial	[199]
Ag	*Oocystis*	10 to 20 ^b^	AgNO_3_	Yes	No	Antimicrobial	[199]
Ag	*Parachlorella*	12 ^a^	AgNO_3_	No	No	NS	[200]
Ag	*Spirogyra*	50 to 114	AgNO_3_	Yes	No	Antimicrobial Insecticidal Antioxidant	[201]
Ag	*Spirulina*	9.0 ^b,c^	AgNO_3_	Yes	No	Antimicrobial	[189]
Fe	*Spirulina*	<10	FeCl_3_	No	No	Photocatalytic	[202]
Ti	*Phaeodactylum*	50 to 130	Ti(OH)_2_	Yes	Yes	Cytotoxicity Antimicrobial	[203]

* Metal element from the obtained nanoparticles: in metal oxide nanoparticles and other nanoparticles (e.g., chloride or sulfide), the non-metallic elements are omitted (e.g., O, Cl, S). NS: not studied; ^a^ Mean value; ^b^ Most nanoparticles; ^c^ Measured with atomic force microscopy (AFM).

In fact, most of the studies compiled in this review not only synthesize and characterize the MNPs but also test at least one of their properties (91%), which reveals the wide interest in the practical application of the synthesized NPs. Among these studies, 73% test their antimicrobial properties, 23% their cytotoxicity, 18% their photocatalytic capacity, 14% their antioxidant capacity and 11% their antibiofilm potential. A few studies also test other properties, such as their biocidal properties (insecticidal, larvicidal, acaricidal, antialgal, antiamoebic and antiparasitic), their phytotoxicity, their detoxification potential, their application for UV protection and their anti-inflammatory capacity. The antimicrobial properties of the NPs were tested on more than 30 species of bacteria and more than 10 species of fungi, mostly pathogenic (73%) either to humans (61%) or plants (12%).

Additionally, the characterization of NPs was performed in all studies. While more than 20 different techniques were used to characterize the NPs, 4 of them were employed across most studies. UV-Vis was used to confirm the synthesis of MNPs (94% of studies) by detecting the presence of absorption bands associated with their surface plasmon characteristics. TEM was employed to visualize and determine the morphology and size of the NPs (85% of studies). XRD analysis of the NPs enabled the elementary determination of the NPs, as well as of their crystalline structure (78% of studies). Finally, to determine the functional groups associated with the capping molecules covering their surface, FTIR was used (83% of studies). Other methods, either complementary to the previous ones or used alternatively, were also employed. SEM (50% of studies) was used to determine the topographic morphology and size of NPs. EDX (38% of studies) was used complementarily to either SEM or TEM to determine the elemental composition of the NPs. Lastly, DLS was used to determine the hydrodynamic diameter of the NPs (40% of studies). Additionally, two relevant parameters—the zeta-potential determination, which is associated with the aggregation potential and consequently with dispersity and stability over time, and the yield of reaction, which is associated with the efficiency of the reaction—were reported in 35% and 11% of the studies, respectively.

## 4. Antimicrobial Activity of Metal Nanoparticles

### 4.1. Antimicrobial Mechanisms—State of the Art

One of the main reasons behind the growing interest in the study of MNPs synthesized using molecules of biological origin is the strong evidence of their potential antimicrobial activity. In particular, this is because they seem to be good candidates for use against MDRM, either as conventional antimicrobial adjuvants or even as their substitutes [204]. However, despite their known properties, the molecular mechanisms behind their antimicrobial activity are not fully determined or understood. In an attempt to answer this question, in the last decade, a vast number of studies have been conducted [205,206,207,208,209,210,211,212,213,214,215,216,217,218,219,220,221,222,223,224,225,226,227,228,229,230,231,232,233,234,235,236,237,238,239,240,241,242]. In these studies, mostly AgNPs (72% of studies) but also other metal and metal oxide NPs, such as zinc oxide nanoparticles (ZnONPs) (10%) and copper nanoparticles (CuNPs) (5%)—and also gold, iron oxide, magnesium oxide, manganese and titanium nanoparticles (AuNPs, FeONPs, MgONPs, Mn and TiO_2_NPs) (3% each)—were used against Gram-negative (77%) and Gram-positive (38%) bacteria and against fungi (5%).

Despite some disagreement [205,206], most studies determined that the antimicrobial mechanism of MNPs involves, at some point, the formation of reactive oxygen species (ROS) [207,208,209,210,211,212,213,214,215,216,217,218,219,220,221,222,223,224] and intracellular content leakage due to cell membrane disruption [208,209,210,211,212,213,214,215,216,217,218,219,220,221,224,225,226,227,228,229,230,231,232]. However, the reasons behind these occurrences and the order in which they occur differ across studies. Many also agree that metallic ions (M^x^) availability inside cells is important [206,207,209,211,213,215,217,223,228,229,233,234,235,236,237], with some showing that M^x^ extracellular sequestration by extracellular polymeric substance (EPS) decreases the antimicrobial activity of MNPs [225,236]. This interaction with EPS might also hinder biofilm formation and be the reason behind some MNPs antibiofilm properties [208,224]. Nevertheless, comparisons between M^x^ and MNPs antimicrobial activity revealed lower antimicrobial activity from extracellular M^x^ than MNPs. These results evidenced differences between the mechanisms of these species regarding both efficacy and effects [209,211,215,216,238]. These differences can probably be explained by the different mechanisms that lead to the cell uptake of M^x^ in each case. Extracellular M^x^ uptake seems to be dependent on membrane protein ion transporters [206,209], which constrains its intracellular availability and does not disrupt the cell membrane [209]. On the other hand, M^x^ from MNPs seems to be directly released inside the cells upon MNPs contact with cell surface due to the disruption of the cell outer membrane. This disruption may be explained by several mechanisms that follow the electrostatic interaction between the positively charged MNPs and negatively charged cell membranes [215]. This interaction may lead to conformational changes in membrane phospholipases (namely, phospholipase A), which promotes phospholipid hydrolysis, ROS formation and lipid peroxidation, all culminating in the formation of pores [209,215,216]. These processes may lead to cell membrane depolarization, which results in K^+^ leakage [230,235]. MNPs have also been shown to disrupt cell membrane redox potential either upon M^x^ interaction with membrane ATPases (proton fluxes decrease) [226] or through disruption of the respiratory chain [210,229] by direct (e.g., dehydrogenase [232]) or indirect (e.g., GAPDH, TCA or PPP enzymes [209,211]) enzymatic inactivation [205,234]. In fact, many studies evidence the importance of M^x^ interaction and inhibition of several metabolic enzymes and propose this as the primary step of MNPs intracellular mechanism, which triggers all the other metabolic effects [205,209,211,216,220,234,239]. Ultimately, the disruption of the respiratory chain results in intracellular ROS content increase, which induces oxidative stress [214,221,222,223], protein damage [223] and DNA disruption [214,219,220,229]. Additionally, studies where the inhibition of antimicrobial MNPs activity by antioxidants [219,230] and an increase in glutathione peroxidase activity [213] have been observed highlight the importance of ROS and oxidative stress to their antimicrobial mechanism [219,230]. Moreover, “omics” approaches show that MNPs mechanism leads to the overexpression of stress-related proteins [208,222], and studies with microbial strains knockout for genes associated with metal efflux pumps, protein damage repair and oxidative stress protection [223] further support this hypothesis. Finally, explanations based on the internalization of intact MNPs were disproved by studies, which showed that immobilized MNPs also showed high antimicrobial activity [209,215,218,220,225,240], sometimes even higher than colloidal MNPs [209,215,240].

Moreover, it has been determined that antimicrobial activity is highly influenced by the MNPs’ surface molecules (capping agents) and by the crystalline phases of the MNPs [241], which supports the hypothesis that MNPs contact with cell surface is an important step of the antimicrobial mechanism [209,211,212,215,221,227,228,240]. This hypothesis also helps explain the differences in antimicrobial activity between microorganisms with different cell surface properties [213,215,221,226,227,229,231,235,238,239,240,242] because these variations can either help or hinder the interaction of cell surface molecules with MNPs’ capping agents. 

### 4.2. Evidence-Based Proposed Antimicrobial Mechanisms

**Figure 2:**
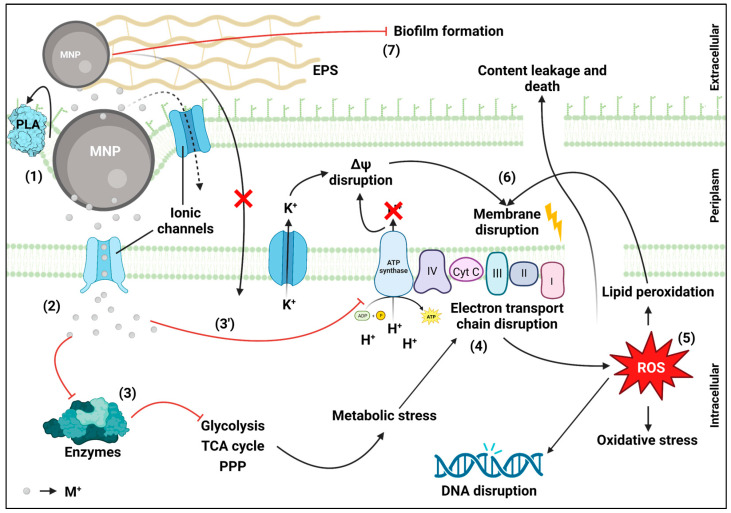
Metal nanoparticles antimicrobial mechanisms. (1) MNPs interaction with the outer membrane leading to PLA activation, phospholipid hydrolysis and formation of pores; (2) M^+^ release into the periplasm and diffusion to cytoplasm through ionic channels; (3) enzyme inhibition from interaction with M^+^ leading to metabolic stress induction; (3′) respiratory chain disruption by ATPase disturbance (direct disruption); (4) respiratory chain indirect disruption; (5) ROS content increase resulting in DNA disruption and oxidative stress induction; (6) membrane disruption from ROS-induced lipid peroxidation resulting in the formation of pores and consequent cell content leakage leading to cell death; (7) MNPs interaction with EPS hindering biofilm formation. PLA: outer membrane phospholipase; TCA cycle: Tricarboxylic acid cycle; PPP: Pentose phosphate pathway; ROS: reactive oxygen species. Created with BioRender.com.

Based on the analysis of the results presented above, a general antimicrobial mechanism is proposed (Figure 2). In sum, MNPs contact with the cell surface leads to membrane morphological changes activating membrane phospholipases that disrupt the outer membrane structure, which leads to the formation of pores. Then, the spontaneous and enzyme-catalyzed oxidation of MNPs releases M^x^, which enter into the cell plasma membrane through ionic protein transporters. Once inside the cells, M^x^ interact with several enzymes inhibiting them, which leads to a metabolic cascade effect that ultimately results in the increase in intracellular ROS concentration, culminating in oxidative and metabolic stress induction, intracellular content leakage and cell death.

## 5. Potential Use of Metal Nanoparticles in Cultural Heritage Conservation

In the last couple of decades, MNPs have been widely studied, aiming toward their application in several scientific branches [8]. Their photodegradation properties applied to toxic molecules, as well as their antimicrobial properties, both supposedly associated with their own biocompatibility, have given rise to high scientific interest and production in the fields of detoxification and pharmaceutics, respectively. 

Despite being a small fraction when compared with studies on the previous fields, in recent decades, the application of MNPs to cultural heritage materials due to their properties (cleaning [243,244,245], hydrophobicity [244,246], photocatalytic [244,247,248,249], consolidation [250,251,252,253,254], deacidification [243,255,256] and antimicrobial activity [257,258,259,260,261,262,263,264,265,266,267,268,269,270,271,272]) has also started to elicit interest (Table 4).

### 5.1. Metal Nanoparticles Application on Materials Used in Cultural Heritage

Bellow, thirteen publications where MNPs antimicrobial activity has been studied against bacteria (nine studies), fungi (nine studies) and microalgae (one study) growth on different materials (six on stone, four on paper, two on textiles and one on wood) used in cultural heritage are presented, as well as the summary of their main results. Most of these studies used MNPs that were synthesized either via chemical, physical or a combination of both methods. Four studies [260,261,272,274] used biosynthesized MPs. 

Studies where MNPs antimicrobial activity was tested in vitro (in culture medium rather than materials) against microorganisms collected from cultural heritage contaminated materials are mentioned in Section 5.2.

#### 5.1.1. Stone

Bellissima et al. [259] tested the antimicrobial activity of AgNPs (70 nm) grafted on *Pietra Serena* against *Bacillus subtilis*. Treated stone samples presented 50% to 80% reduction in cell viability (CFU) compared with controls, the best results being obtained using AgNPs at 6.7 μg/cm^2^. Carrillo-González et al. [261] tested the effect of AgNPs (<100 nm)—synthetized using two plant extracts—on stucco and samples extracted from an ancient city stone wall (calcite and basalt) against *Pectobaterium carotovorum* and *Alternaria alternata* isolated from the same place. Their results showed that AgNPs are effective as both preventive (74%) and corrective (61%) treatment against *P. carotovorum*, while they only hindered *A. alternata* growth when applied as correctives (95%). The hypothesis of the type of stone influencing the effects of AgNPs was also raised. Sierra-Fernandez et al. [262] tested the effect of MgONPs (20 nm) and ZnONPs (97 nm) against *Aspergillus niger*, *Penicilium oxalicum*, *Paraconiothyrium* sp. and *Pestalotiopsis maculans* growth in dolostone and limestone. MgONPs presented antifungal activity, with a MIB of 1.25 mg/mL for all fungi except *Pestalotiopsis maculans*, which have MIB value of 5 mg/mL. ZnONPs did not present antifungal activity in all concentrations tested (max. 10 mg/mL). Becerra et al. [263] tested the effects of silver (5–6 nm), copper (40–60 nm), zinc oxide (50 nm) and titanium dioxide (25 nm or 85 nm) on microalgal and cyanobacteria development in stone (limestone). All NPs but TiO_2_ showed great effectiveness in preventing fouling by microalgae and cyanobacteria, with AgNPs being the most effective (78% effectiveness). Mu et al. [258] studied the antimicrobial activity of chemically synthesized AgCl-ZnO nanoclusters (50-100 nm) against *Bacillus subtilis*, *Escherichia coli* and *Aspergillus niger* on stones previously immersed in an AgCl-ZnO nanocluster dispersion. The comparison between treated and untreated stones showed a significant reduction in both bacterial and fungal growth. Li et al. [260] studied the antimicrobial activity of ZnONPs (3–5 nm) in vitro and grafted on sandstone samples against *Escherichia coli*, *Micrococcus yunnanensis* and *Aspergillus* sp. The results showed that ZnONPs (10 mg/mL) completely inhibited bacterial growth (100%) and significantly inhibited fungal growth (~50%) in vitro, while fluorescence microscopy images confirmed that immobilized ZnONPs exhibited antimicrobial and antibiofilm activity. 

#### 5.1.2. Paper

Fouda et al. [264] tested the effects of silver and ZnONPs (size not reported) on microorganisms isolated from poorly stored archaeological manuscripts, which resulted in total inhibition of the growth of *Bacillus subtilis* using 1 mM of both AgNPs and ZnONPs. The growth of *Penicillium chrysogenum* using 2 mM AgNPs and ZnONPs also resulted in high inhibition (90%). Castillo et al. [265] tested the effects of MgONPs (12 nm; 0.86 mg/g) on archaeological 18th century paper against *Aspergillus niger*, *Cladosporium cladosporioides* and *Trichoderma reesei*. Fungicidal properties of MgO (10 mg/mL) were achieved in *Aspergillus niger* and *Trichoderma reese*, while in *Cladosporium cladosporioides*, they only presented fungistatic properties (10 mg/mL). Castillo et al. [266] also tested the effect of MgONPs (10 nm) on archaeological 18th century paper against *Escherichia coli* and *Bacillus subtilis*, which showed a minimum bactericidal concentration (MBC) of 1.5 mg/mL and 0.75 mg/mL, respectively. In another study, Fouda et al. [272] also tested the effects of Ag (26–62 nm) and ZnO (9–23 nm) NPs biosynthesized in *Penicillium chrysogenum* culture supernatants using silver nitrate and zinc acetate, respectively, against *Aspergillus niger* strains previously isolated from “*Description de l’Égypte*”, an archaeological manuscript from the 19th century. Paper filter samples previously treated with 2 mM MNPs (AgNPs and ZnONPs) showed total growth inhibition 7 days after *A. niger* inoculation, which decreased slightly to 97% and 98% after 21 days for AgNPs and ZnONPs, respectively.

#### 5.1.3. Textile

Pietrzak et al. [267] tested the effects of AgNPs (10–80 nm) on archaeological textiles (wool, cotton and sisal) against 15 bacterial and 3 fungal strains. The results showed high variability in effectiveness (31–100%) depending on the species. *Bacillus* spp. were more resistant, while growth inhibition was higher against *Oceanobacillus* sp., *Kocuria* sp., *Paracoccus* sp., *Cladosporium* sp. and *Penicilium* sp. Eskani et al. [268] tested the effect of ZnONPs (55 nm) on a traditional cotton fabric from Indonesia (batik) against *Staphylococcus aureus* and observed a high antibacterial effect (75% of chloramphenicol).

#### 5.1.4. Wood

De Filpo et al. [269] tested the effect of TiO_2_NPs (50 nm) on eight types of wood used in cultural heritage against *Hypocrea lixii* (white rot) and *Mucor circinelloides* (brown rot), which inhibited their growth.

### 5.2. Metal Nanoparticles’ Antimicrobial Activity against Microorganisms Collected from Contaminated Cultural Heritage Materials

Since biosynthesized MNPs mechanisms and effects on both microorganisms and materials are not well determined, and given that cultural heritage objects are usually priceless and irreplaceable, their conservation using direct application of MNPs is not usually accepted or desirable (especially for research purposes). Thus, most of the previously discussed studies used samples from materials equivalent to the real ones. However, the microorganisms whose growth is tested on these samples might not be cultural heritage contaminants. Research on MNPs treated samples from materials used in cultural heritage against microorganisms collected from contaminated cultural heritage objects should also be conducted. However, other approaches also produce valuable information. 

Instead of testing the antimicrobial activity of MNPs directly on the materials used in cultural heritage, a few studies [257,270,271] tested it in vitro (i.e., in culture media) against microorganisms collected and isolated from contaminated cultural heritage materials. This approach, when integrated with the ones mentioned above, might give information regarding the possible effect of the support (i.e., material) on the antimicrobial activity of MNPs. Their results are summarized below.

Gutarowska et al. [270] tested the effect of commercially available colloidal AgNPs (10–80 nm) on 32 microbial strains (15 bacteria, 3 yeasts and 14 molds) collected from cultural heritage materials and surfaces from their storage rooms. Their results showed that with a concentration of 45 ppm, the AgNPs tested were effective bactericides against 94% of the microorganisms studied. Gambino et al. [271] tested the effect of ZnONPs (30–70 nm) against *Alternaria alternata*, *Aspergillus niger*, *Penicillium chrysogenum* and *Penicillium pinophilum* from ancient Egyptian paintings from tombstones. *P. pinophilum* was the most sensitive one, showing 57% and 68% growth reduction (culture diameter in agar plate) with a concentration of ZnONPs of 0.125% and 0.25%, respectively. *P. chrysogenum* had 36% and 39% growth reduction under the same conditions. *A. alternata* and *A. niger* growth was reduced by 27% and 13%, respectively, with both 0.125% and 0.25% concentration. The effect of ZnONPs on biofilm growth reduction in *P. pinophilum, P. chrysogenum* and *A. alternata* was also observed by Ref. [271]. De la Rosa-García et al. [257] tested the in vitro effects of CaZn_2_(OH)_6_ NPs (calcium zincate NPs) (~43 nm) on mold strains isolated from limestone and dolostone walls (*Aspergillus niger*, *Cladosporium cladosporioides*, *Curvularia lunata*, *Penicillium oxalicum*, *Pestalotiopsis maculans*, *Phoma eupyrena* and *Scolecobasidium constrictum*—limestone; *Gliomastix* sp., *Penicillium* sp. and *Ramichloridium* sp.—dolostone). The authors reported the ability of CaZn_2_(OH)_6_ NPs to inhibit microbial growth and, despite substantial variability, their results revealed fungicidal effects on all microorganisms tested, with minimal fungicidal concentrations ranging between 156 and 1250 mg/mL. Abdel-Maksoud et al. [274] studied the in vitro antimicrobial potential of AgNPs and MgONPs biosynthesized using *Aspergillus niger* and *Rhizopus oryzae*, respectively, against fungi isolated from an archaeological skeleton from the Greco-Roman period (*Aspergillus flavus*, *Aspergillus delicatus*, *Aspergillus parasiticus*, *Aspergillus niger*, *Aspergillus oryzae*, *Penicillium expansum*, *Penicillium oxalicum*, *Penicillium digitatum*, *Cladosporium* sp. and *Paecilomyces* sp.). AgNPs (3–13 nm) and MgONPs (8.0–47.5 nm) applied with a concentration of 300 ppm inhibited fungal growth by 69.1–82.5% and 59.5–74.3%, respectively, depending on the microorganism, which showed good potential for their future application.

Analysis of the results from the previously mentioned studies evidenced several aspects related to the antimicrobial activity of MNPs against microorganisms isolated from cultural heritage materials. The results from Ref. [270] revealed that yeasts, with an average minimum fungicidal concentration (MFC) of 22.5 ppm, seem to be more susceptible to AgNPs compared to bacteria (average minimum bactericidal concentration (MBC) of 28.1 ppm) and molds (average MFC of 39.4 ppm). Interestingly, Gram-negative bacteria (average MBC of 16.9 ppm) are the most susceptible microorganisms tested, while Gram-positive cocci (average MBC of 36.0 ppm and one resistant strain (MBC > 45 ppm)) and Gram-positive endospore-forming rods (average MBC of 26.5 ppm and four out of nine resistant strains) are the least susceptible microorganisms tested. These observations seem to point toward a higher resistance to MNPs of multicellular microorganisms relative to unicellular ones—apart from Gram-positive bacteria whose thick cell wall might hinder the MNPs effect [275]. Elemental composition differences are evidenced by studies on Zn-based NPs use against *Aspergillus niger*. Although within the same size range (~43 nm and 30–70 nm), one study used CaZn2(OH)6NPs [257] while another used ZnONPs [271]. The first one obtained an MFC of 156 ppm, while the second only reduced *Aspergillus niger* growth by 13% with a concentration of 2500 ppm. These differences might possibly be explained by distinct interaction of the MNPs with the outer membrane of the cells and their ability to release M^x^ [275]. Additionally, the size of the MNPs seems to be inversely correlated with their antimicrobial potential, with smaller MNPs apparently being more effective than larger ones [274]. Overall, these studies show promising results regarding the antimicrobial potential of MNPs against cultural heritage microbial contaminants, which are further supported by the results presented in Section 5.1.

## 6. Closing Remarks and Future Research Guidelines

The available research studies seem to show that the biosynthetic mechanism of MNPs is highly dependent on the reaction media, which influences their morphology and consequently their properties, which also change due to variations in their capping agents. These studies also evidence the involvement of different molecules in the mechanism—not only enzymes, as previously thought—and suggest that the sole requirement for MNPs biosynthesis is the presence of molecules with reducing capacity (e.g., molecules with hydroxyl groups next to electron-stabilizing groups). Nevertheless, in order to assess the mechanism of microbial inhibition, since FTIR (the most used technique to ascertain the molecules involved in the mechanism) does not enable specific molecules’ identification, studies using more sensitive and specific techniques (e.g., MS) are needed. Moreover, studies on the relative impact of different molecules would also contribute to the determination of the mechanism. 

Regarding studies that use microbial cultures for biosynthesis of MNPs with extracellular metabolites, there are a few concerns. Firstly, many studies report the synthesis of MNPs while showing images of the agglomerates. It is important to accurately determine whether the NPs present in those agglomerates are bound or just stacked (e.g., using DLS). Furthermore, most studies do not report the yield of the reaction. The determination and reporting of this parameter are of utmost importance to compare the methodologies and microbial cultures—even more when the global goal seems to be their alternative use in chemical synthesis. Finally, more studies using fungi and microalgae cultures are needed, especially knowing that the ones available show promising results, such as biosynthesis of smaller MNPs, which seem to be related to more efficient antimicrobial activity.

Variability in the cellular structures, intracellular compartmentalization and metabolism between microorganisms (e.g., Gram positive vs. Gram negative; prokaryotes vs. eukaryotes) may result in changes in the mechanism of antimicrobial activity of MNPs (e.g., Gram-positive bacterial wall may hamper the availability of MNPs to reach the plasma membrane, lowering M^x^ intracellular availability), which probably explains the diverse effectiveness of the same MNPs on distinct microorganisms. Additionally, MNPs from different metal elements have distinct properties that influence their effects on microorganisms (e.g., the oxidation state of the metal ion may change its ability to interact with and inhibit an enzyme), which may also contribute to the latter. Notwithstanding, based on the previously presented research, a general mechanism of MNPs interactions with microorganisms was proposed, which is dependent on several crucial steps: MNPs contact with cell surface, M^x^ intracellular availability, M^x^ enzyme and DNA disruption, ROS formation, membrane destruction and content leakage. The associations between MNPs morphology and capping agents have also been shown to influence their antimicrobial activity. Future research on the determination of the antimicrobial mechanism needs to employ more precise techniques and methodologies, which not only quantify molecules but also localize them in space and time (e.g., fluorescence microscopy).

Finally, despite few studies having been performed on the application of MNPs to cultural heritage materials, the ones available show promising results. However, being a recently emergent field, the biosynthesis of MNPs is still in an early development stage, with insufficient knowledge regarding its mechanisms and antimicrobial activity not being consensual. In addition, MNPs size and capping agents using biosynthesis are highly variable and difficult to control. Possibly for these reasons, studies using biosynthesized MNPs are severely lacking, with most researchers choosing to test their own chemically or physically synthesized MNPs or even commercially available ones, whose sizes and capping agents are easier to control or even predetermine. The number of studies on MNPs application to non-stone materials is also low. Research focused on diverse cultural heritage materials is needed. Future research should also focus on accurately determining the impact of MNPs on cultural heritage materials, especially studying MNPs stability over long periods.

## Figures and Tables

**Table 1:** Biosynthesis of metal nanoparticles using bacteria and archaea. Several examples of literature published in the last five years (2018–2022). The microbial genera, the metal and its precursor and the size of the metal nanoparticles obtained are presented. It is also stated whether the antimicrobial activity and toxicity of the nanoparticles were tested and which properties were found.

Metal *	Microbial Genera	NPs Size (nm)	Precursor	Antimicrobial Activity Studies	Toxicity Studies	Main Properties	Ref.
Bacteria							
Ag	*Acinetobacter*	11 to 9	AgNO_3_	Yes	No	Antimicrobial	[51]
Ag	*Actinokineospora*	19 to 41	AgNO_3_	No	Yes	Mosquitocidal	[52]
Ag	*Amycolatopsis*	35 ^c^	AgNO_3_	Yes	No	Antimicrobial	[53]
Ag	*Arthrobacter*	12 to 50	AgNO_3_	Yes	No	Antimicrobial	[54]
Ag	*Bacillus*	11 to 39	AgNO_3_	No	Yes	Photocatalytic	[55]
Ag	*Bacillus*	18 to 39	AgNO_3_	Yes	Yes	Antimicrobial	[56]
Ag	*Bacillus*	10 to 20	AgNO_3_	Yes	No	NS	[57]
Ag	*Bacillus*	20 to 40	AgNO_3_	No	Yes	Photocatalytic Cytotoxicity	[58]
Ag	*Bacillus*	25 to 70	AgNO_3_	Yes	Yes	Antimicrobial Antibiofilm Cytotoxicity	[59]
Ag	*Bacillus*	20 to 60	AgNO_3_	Yes	No	Antimicrobial Antioxidant Photocatalytic	[60]
Ag	*Bacillus*	10 to 30	AgNO_3_	Yes	Yes	Photocatalytic Cytotoxicity Antimicrobial Antibiofilm	[61]
Ag	*Bacillus*	1.5 to 8.8	AgNO_3_	Yes	Yes	Antimicrobial Cytotoxicity	[62]
Ag	*Bacillus*	1.8 to 12.4	AgNO_3_	No	No	Photocatalytic	[63]
Ag	*Bacillus*	3 to 20	AgNO_3_	Yes	No	Antimicrobial	[64]
Ag	*Bacillus*	65 to 70	AgNO_3_	Yes	No	Antimicrobial	[65]
Ag	*Bacillus*	3 to 15	AgNO_3_	Yes	No	Antimicrobial	[66]
Ag	*Bacillus*	10 to 33	AgNO_3_	Yes	Yes	Antimicrobial Mosquitocidal	[67]
Ag	*Bacillus*	6 to 50	AgNO_3_	Yes	Yes	Antimicrobial Cytotoxicity Larvicidal	[68]
Ag	*Bacillus*	5 to 7.1	AgNO_3_	Yes	No	Antimicrobial Antioxidant Photocatalytic	[69]
Ag	*Bacillus*	13 to 50	AgNO_3_	Yes	No	Antimicrobial	[70]
Ag	*Citrobacter*	5 to 15	AgNO_3_	Yes	No	Antimicrobial	[71]
Ag	*Deinococcus*	5 to 16	AgNO_3_	No	No	NS	[72]
Ag	*Desertifilum*	6.2 to 11.4	AgNO_3_	Yes	No	Antimicrobial Antioxidant	[73]
Ag	*Enterobacter*	15 to 46	AgNO_3_	No	No	NS	[74]
Ag	*Enterococcus*	10 to 16	AgNO_3_	Yes	No	Antimicrobial Antioxidant	[75]
Ag	*Escherichia*	10 to 16.7	AgNO_3_	No	Yes	Cytotoxicity	[76]
Ag	*Escherichia*	6 to 17	AgNO_3_	Yes	No	Antimicrobial	[77]
Ag	*Flavobacterium*	10 to 24	AgNO_3_	Yes	Yes	Antimicrobial Antioxidant Cytotoxicity	[78]
Ag	*Labrenzia*	14.0 to 37.0	AgNO_3_	No	No	NS	[79]
Ag	*Lactobacillus*	30 to 100	AgNO_3_	Yes	Yes	Antimicrobial Antioxidant Cytotoxicity	[80]
Ag	*Lactobacillus*	31 to 100	AgNO_3_	Yes	No	Antimicrobial	[81]
Ag	*Leclercia*	18 to 39	AgNO_3_	Yes	No	Antimicrobial Photocatalytic Antibiofilm	[82]
Ag	*Lysinibacillus*	8 to 30	AgNO_3_	Yes	No	Antimicrobial	[83]
Ag	*Lysinibacillus*	14 to 21	AgNO_3_	Yes	Yes	Antimicrobial	[84]
Ag	*Massilia*	15 to 55	AgNO_3_	Yes	No	Antimicrobial	[85]
Ag	*Methylophilus*	38.9 ^a^	AgNO_3_/ [Ag(NH_3_)_2_]NO_3_	Yes	No	Antibiofilm	[86]
Ag	*Nostoc*	6 to 45	AgNO_3_	Yes	No	Antioxidant Antimicrobial	[87]
Ag	*Paenarthrobacter*	13 to 27	AgNO_3_	Yes	No	Antimicrobial	[88]
Ag	*Phormidium*	6.5 to 12.2	AgNO_3_	Yes	No	Antimicrobial Antioxidant	[73]
Ag	*Pilimelia*	3 to 36	AgNO_3_	Yes	Yes	Antimicrobial Cytotoxicity	[89]
Ag	*Pseudoduganella*	8 to 24	AgNO_3_	Yes	No	Antimicrobial	[90]
Ag	*Pseudomonas*	7.27 ^a^	AgNO_3_	Yes	No	Antimicrobial	[91]
Ag	*Pseudomonas*	2.4 to 53.5	AgNO_3_	Yes	Yes	Antimicrobial Antioxidant	[92]
Ag	*Pseudomonas*	11 to 25	AgNO_3_	Yes	Yes	Antimicrobial Cytotoxicity	[93]
Ag	*Rhizopus*	6 to 40	AgNO_3_	Yes	Yes	Antimicrobial Cytotoxicity Larvicidal	[68]
Ag	*Shewanella*	19 to 73	AgNO_3_	Yes	Yes	Antimicrobial Cytotoxicity	[94]
Ag	*Solibacillus*	70 to 130	AgNO_3_	Yes	No	Antimicrobial Antibiofilm	[95]
Ag	*Sphingobium*	7 to 22	AgNO_3_	Yes	No	Antimicrobial	[85]
Ag	*Stenotrophomonas*	5 to 30	AgNO_3_	No	Yes	Phytotoxicity	[96]
Ag	*Streptomyces*	11 to 62	AgNO_3_	Yes	No	Antimicrobial Antioxidant Larvicidal	[97]
Ag	*Streptomyces*	64 ^a,b^	AgNO_3_	Yes	No	Antimicrobial Photocatalytic	[98]
Ag	*Streptomyces*	13.9 to 35.1 ^a^	AgNO_3_	No	No	NS	[99]
Ag	*Streptomyces*	16.4 ^a^	AgNO_3_	Yes	No	Antimicrobial	[100]
Ag	*Streptomyces*	5 to 22	AgNO_3_	Yes	Yes	Antimicrobial	[101]
Ag	*Streptomyces*	19.0 to 32.1	AgNO_3_	Yes	Yes	Antimicrobial Cytotoxicity	[102]
Ag	*Streptomyces*	11 to 30	AgNO_3_	Yes	No	Antimicrobial Antibiofilm Larvicidal	[103]
Ag	*Streptomyces*	6 to 30	AgNO_3_	Yes	Yes	Antimicrobial Cytotoxicity Larvicidal	[68]
Ag	*Streptomyces*	40 to 100	AgNO_3_	Yes	Yes	Antimicrobial Cytotoxicity	[104]
Ag	*Terrabacter*	6 to 24	AgNO_3_	Yes	No	Antimicrobial	[105]
Ag	*Thiosphaera*	5 to 51	AgNO_3_	Yes	Yes	Cytotoxicity Antimicrobial	[106]
Ag	*Vibrio*	32.7 to 107.2 ^b^	AgNO_3_	Yes	No	Antimicrobial Antibiofilm	[107]
Au	*Amycolatopsis*	44.4 ^a,b^	HAuCl_4_	Yes	Yes	Antimicrobial Antibiofilm Antioxidant Cytotoxicity	[108]
Au	*Citricoccus*	25 to 65	HAuCl_4_	No	No	NS	[109]
Au	*Leuconostoc*	47.77 ^a,b^	HAuCl_4_	Yes	No	Antimicrobial Antibiofilm	[110]
Au	*Nocardiopsis*	7 to 15	HAuCl_4_	Yes	Yes	Antimicrobial Antioxidant Cytotoxicity	[111]
Au	*Paracoccus*	20.93 ^a^	HAuCl_4_	No	Yes	Antioxidant Cytotoxicity	[112]
Au	*Streptomyces*	12.2 ^a^	HAuCl_4_	Yes	No	Antimicrobial Antibiofilm	[113]
Cu	*Bacillus*	10 to 70	CuSO_4_	Yes	No	Antimicrobial	[114]
Cu	*Brevundimonas*	20 to 80	CuCl_2_	Yes	No	Antimicrobial	[115]
Cu	*Brevundimonas*	10 to 70	CuSO_4_	Yes	No	Antimicrobial	[114]
Cu	*Klebsiella*	19 to 47	CuSO_4_	No	Yes	NS	[116]
Cu	*Lactobacillus*	30 to 75	CuSO_4_	Yes	Yes	Antimicrobial Cytotoxicity	[117]
Cu	*Marinomonas*	10 to 70	CuSO_4_	Yes	No	Antimicrobial	[114]
Cu	*Pseudomonas*	10 to 70	CuSO_4_	Yes	No	Antimicrobial	[114]
Cu	*Rhodococcus*	10 to 70	CuSO_4_	Yes	No	Antimicrobial	[114]
Cu	*Shewanella*	4 to 10	CuCl_2_	No	No	Photocatalytic	[118]
Cu	*Streptomyces*	1.5 to 8.5	CuSO_4_	Yes	Yes	Antimicrobial	[119]
Cu	*Streptomyces*	1.7 to 13.5	CuSO_4_	Yes	No	Antimicrobial Antioxidant	[120]
Cu	*Streptomyces*	13 to 35	CuSO_4_	Yes	No	Antimicrobial Photocatalytic Antibiofilm	[121]
Fe	*Bacillus*	60 to 80	Fe_2_O_3_	No	Yes	Antioxidant	[122]
Fe	*Bacillus*	98.17 ^b^	FeCl_2_/FeCl_3_	No	No	Photocatalytic	[123]
Fe	*Bacillus*	53.5 ^b^	FeCl_2_/FeCl_3_	No	No	Photocatalytic	[123]
Fe	*Bacillus*	37.4 ^b^	FeCl_2_/FeCl_3_	No	No	Photocatalytic	[123]
Fe	*Streptomyces*	65.0 to 86.7	FeCl_2_/FeCl_3_	Yes	Yes	Antioxidant Antimicrobial Cytotoxicity	[124]
Ti	*Pseudomonas*	6.83 ^a^	Ti(OBu)_4_	Yes	No	Antimicrobial	[91]
Zn	*Alkalibacillus*	1 to 30	ZnSO_4_	No	No	NS	[125]
Zn	*Arthrospira*	30 to 55	Zn(CH_3_COO)_2_	Yes	Yes	Antimicrobial Cytotoxicity	[126]
Zn	*Bacillus*	22 to 59	Zn(NO_3_)_2_	Yes	No	Antimicrobial	[127]
Zn	*Bacillus*	35 to 90	Zn(NO_3_)_2_	Yes	No	Antimicrobial Antibiofilm	[127]
Zn	*Bacillus*	16 to 25	ZnSO_4_	No	No	NS	[128]
Zn	*Bacillus*	16 to 20	Zn(NO_3_)_2_	No	Yes	Phytotoxicity	[129]
Zn	*Escherichia*	6 to 19	Zn(NO_3_)_2_	Yes	No	Antimicrobial	[130]
Zn	*Lactobacillus*	30 ^a^	Zn(NO_3_)_2_	Yes	No	Antimicrobial	[131]
Zn	*Paenibacillus*	56 to 110	ZnO	Yes	No	Antimicrobial	[132]
Zn	*Streptomyces*	37.9 ^a^	Zn(CH_3_COO)_2_	Yes	Yes	Antimicrobial	[133]
Archaea							
Fe	*Halobiforma*	25 ^a^	FeSO_4_	No	No	NS	[134]

* Metal element from the obtained nanoparticles: in metal oxide nanoparticles and other nanoparticles (e.g., chloride or sulfide), the non-metallic elements are omitted (e.g., O, Cl, S). NS: not studied; ^a^ Mean value; ^b^ Measured with dynamic light scattering (DLS); ^c^ Most nanoparticles.

**Table 4:** Metal nanoparticles applied to cultural heritage materials (examples).

Material	Metal Nanoparticles	Properties	References
Stone	Ag	Antimicrobial	[261]
	Ag ^(i)^	Antimicrobial	[259]
	Ag; Cu; ZnO; TiO_2_	Antimicrobial	[263]
	AgCl-ZnO ^(nc)^	Antimicrobial	[258]
	MgO; ZnO	Antimicrobial	[262]
	ZnO ^(i)^	Antimicrobial	[260]
	ZnO; CaZn_2_(OH)_6_	Anti-phototrophic	[273]
	Ba(OH)_2_	Consolidation	[252]
	MgO	Consolidation	[253]
	ZnO	Consolidation	[251]
	ZnO ^(nc)^	Hydrophobicity	[246]
	TiO_2_	Photocatalytic	[247]
	TiO_2_^(i)^	Photocatalytic	[248]
	TiO_2_-ZnO ^(nc)^	Photocatalytic Hydrophobicity Self-cleaning	[244]
Wall paintings	Ba(OH)_2_	Consolidation	[254]
	Mg(OH)_2_	Consolidation	[250]
Paper	Ag; ZnO	Antimicrobial	[264]
	Ag, ZnO	Antimicrobial	[272]
	MgO	Antimicrobial	[266]
	MgO	Antimicrobial	[265]
	Ag ^(i)^	Cleaning Deacidification	[243]
	Mg(OH)_2_	Deacidification	[255]
	Ba(OH)_2_	Deacidification Consolidation	[256]
Canvas	Ag ^(i)^	Cleaning	[243]
Textile	Ag	Antimicrobial	[267]
	ZnO	Antimicrobial	[268]
	Au	Cleaning Glue removal	[245]
Wood	TiO_2_	Antimicrobial	[269]
	TiO_2_^(nc)^	Antifungal Photocatalytic	[249]

^(i)^ immobilized; ^(nc)^ nanocluster.

## Data Availability

The data presented in this study are available in [27,28,29,30,31,32,33,34,35,36,37,38,39,40,41,42,43,44,45,46] (MNPs biosynthesis mechanisms), [51,52,53,54,55,56,57,58,59,60,61,62,63,64,65,66,67,68,69,70,71,72,73,74,75,76,77,78,79,80,81,82,83,84,85,86,87,88,89,90,91,92,93,94,95,96,97,98,99,100,101,102,103,104,105,106,107,108,109,110,111,112,113,114,115,116,117,118,119,120,121,122,123,124,125,126,127,128,129,130,131,132,133,134,136,137,138,139,140,141,142,143,144,145,146,147,148,149,150,151,152,153,154,155,156,157,158,159,160,161,162,163,164,165,166,167,168,169,170,171,172,173,174,175,176,177,178,179,180,181,182,183,184,185,186,187,188,189,190,191,192,193,194,195,199,200,201,202,203] (recent studies on the biosynthesis of MNPs using microorganisms), [205,206,207,208,209,210,211,212,213,214,215,216,217,218,219,220,221,222,223,224,225,226,227,228,229,230,231,232,233,234,235,236,237,238,239,240,241,242] (MNPs antimicrobial mechanisms), and [243,244,245,246,247,248,249,250,251,252,253,254,255,256,257,258,259,260,261,262,263,264,265,266,267,268,269,270,271,272,273] (MNPs applied to Cultural Heritage materials).
